# Standardized food challenges are subject to variability in interpretation of clinical symptoms

**DOI:** 10.1186/s13601-014-0043-6

**Published:** 2014-11-30

**Authors:** Francine C van Erp, André C Knulst, Yolanda Meijer, Carmelo Gabriele, Cornelis K van der Ent

**Affiliations:** Department of Paediatric Pulmonology and Allergology, Wilhelmina Children’s Hospital, University Medical Centre Utrecht, P O Box 85090, 3508 AB Utrecht, The Netherlands; Department of (Paediatric) Dermatology and Allergology, University Medical Centre Utrecht, Utrecht, The Netherlands

**Keywords:** Allergy, Children, Diagnostics, Food challenge, Food allergy, Peanut, Variability

## Abstract

**Background:**

Food challenge tests are the gold standard in diagnosing food allergy. Guidelines provide scoring systems to classify symptoms during challenge and typically recommend that challenges are considered positive when objective symptoms occur. However, currently no standard criteria for the definition of a positive challenge outcome exists and interpretation of food challenges mainly depends on clinical judgment. This study aims to assess inter- and intra-observer variability in outcomes of routinely performed peanut challenges in children.

**Methods:**

All complete food challenge score sheets of double blind placebo controlled peanut challenges performed in 2008-2010 in an academic hospital were included. Score sheets were reassessed independently by three clinical experts including double reassessment in a subset of score sheets. Inter- and intra-observer variability was evaluated using kappa statistics.

**Results:**

We included 191 food challenge score sheets. Inter-observer agreement on overall challenge outcome was moderate (κ = 0.59-0.65) and was fair (κ = 0.31-0.46) on challenges with symptoms. Intra-observer agreement on overall challenge outcome was good (κ = 0.63-0.77) but was moderate (κ = 0.50-0.60) on challenges with symptoms. Subjective symptoms (oral symptoms, abdominal complaints, food aversion) were significantly associated with disagreement between observers.

**Conclusions:**

We demonstrate that, despite strict adherence to guidelines, there is a considerable amount of variability in reassessment of symptoms recorded on food challenges sheets between and within well trained clinicians, especially when subjective symptoms occur.

## Background

Food challenge tests are the gold standard in diagnosing food allergy [1]. Several guidelines and symptom score sheets exist to classify symptoms during challenge. A food challenge is usually considered positive when clear objective symptoms occur on verum and not on placebo [1-3]. Whenever possible, symptoms are supported and objectified by measuring clinical parameters such as blood pressure, oxygen saturation and lung function tests. However, no standard criteria for the definition of a positive challenge outcome exist and the interpretation of food challenges mainly depends on clinical judgment. Especially when clear objective symptoms are absent, determination of food challenge outcome can be difficult. Clinicians may then take other factors (course and reproducibility of symptoms over time, patient characteristics, a “gut feeling” or lessons learned from previous cases) into account to determine challenge outcome. These factors come along with clinical judgment in general and are not easily standardized nor implemented in guidelines. Until now no data on the diagnostic accuracy of the interpretation of symptoms during food challenge are available. In this study we describe inter- and intra-observer variability in reassessment of the outcome of previous performed standardized food challenges by measuring the agreement on the outcome of food challenge score sheets.

## Methods

All complete Double Blind Placebo Controlled Food Challenges (DBPCFCs) (n = 191) for peanut performed in an academic hospital from 2008-2010 were selected for this study. Data were obtained as part of regular patient care and collected retrospectively from electronic patient records in 2012. Food challenge score sheets were used in strictly anonymous form, according to the code of conduct for medical research approved by the hospital’s Medical Ethical Committee.

The DBPCFC protocol used in this study was described earlier by Flinterman et al. [4] In short, increasing amounts of defatted peanut flour from 0.01 to 3000 mg, were given with time-intervals of 15-30 minutes with randomly dispersed placebo’s. Challenges were performed by a nurse practitioner specialized in food allergy and interpreted under supervision of an allergologist. When symptoms occurred the patient was fully examined and in case of doubt or severe symptoms the allergologist was called to interpret these symptoms. All signs and symptoms observed during DBPCFC were recorded in detail on paper food challenge score sheets including timing and administration of doses by a trained nurse and any abnormalities in vital signs (Table 1). Challenges were discontinued and considered positive in case of persistent objective symptoms or if suggestive subjective symptoms (Oral allergy symptoms (OAS)) occurred at 3 subsequent doses or a severe subjective symptom (abdominal pain/nausea with discomfort) lasted for more than 45 minutes. Symptoms within 15 minutes after a placebo dose were considered as placebo reactions. The three observers were clinical experts in food allergy, regularly interpreted food challenges according to the most recent clinical guidelines [2], had the same criteria for classifying a challenge was positive and worked in close cooperation with each other within an expert centre of food allergy. Observer 1 (a paediatric allergologist) performed food challenges in children for 10 years and supervised included food challenges (2-4 years ago). Observer 2 (a paediatric allergologist in training) performed challenges for more than 5 years. Observer 3 (dermatologist and immunologist) performed food challenges in adults for more than 10 years. Anonymous food challenges score sheets (blinded for patient characteristics, randomization and challenge outcome) were individually administrated to the observers. The observers received 25% duplicated score sheets randomly dispersed with the other score sheets without their knowledge. They were asked to determine and argue DBPCFC outcome as positive, negative or when information was insufficient or doubtful as inconclusive. Agreement between observers was defined as a concordant classification of all three observers. Disagreement was defined as a discordant classification between two or three observers.

**Table 1:** **Example Food challenge score sheet**

**Minutes after start**	**Portion**	**Time (hr)**	**Observations/Symptoms**
**Part 1**			
T= 0	1	10.30	At 10:35 patient reports mild abdominal pain, the pain disappeared spontaneously within 10 minutes.
T=15	2	10.45	-
T=30	3	11.00	Patient does not like the food and eats very slowly.
T=45	4	11.15	-
T=60	5	11.30	-
T=75	6	11.45	-
T=90	7	12.00	At 12:05 mild sneezing (2 times), no other symptoms.
T=105	8	12.15	-
**Part 2**			
T=120	9	12.30	-
T=150	10	13.00	At 13.15 Severe vomiting (1 time). No other symptoms.
T=180	11	NA	-
T=210	12	NA	-
T =240	13	NA	-
**Other comments:** Patient is a very difficult eater, and did not like the food during challenge.			

### Statistics

The kappa statistic (κ) was used to determine intra-observer and inter-observer variability between different pairs of observers on overall challenge outcomes and on individual symptoms in challenges with symptoms respectively. Interpretation of the Kappa value: <0.20 = poor agreement; 0.21-0.40 = fair agreement; 0.41-0.60 = moderate agreement; 0.61-0.80 = good agreement; 0.81-1.0 = excellent agreement [5]. For univariable analyses of the association between type of symptoms and the agreement between observers, the chi-square statistic or univariable logistic regression analysis was used. A p-value <0.05 was considered statistically significant.

## Results

Initial DBPCFC outcome was positive in 88 (46%) and negative in 103 (54%) included challenges. Reactions ranged from Sampson grade 1 to grade 4, only one child showed significant changes in vital signs (tachycardia). Baseline characteristics of children who underwent DBPCFC are shown in Table 2. Agreement of observers with initial challenge outcome ranged from 79% - 87%. Based on the reassessment of score sheets the observers fully agreed on 132 of 191 (69%) DBPCFCs, whether the challenge outcome was positive or negative. In 47 (25%) challenges one observer disagreed with the other two, in 12 (6%) challenges complete disagreement (negative, positive and inconclusive classification) was present. Inconclusive challenge outcome was recorded by different observers in 58 (10%) reassessments. Reasons reported for inconclusive judgment were insufficient information (50%), nonspecific symptoms (47%) or unknown (3%). Overall 111 (58%) score sheets could be used to assess inter-observer agreement on individual symptoms. On the remaining 80 (42%) food challenge score sheets no symptoms were reported.

**Table 2:** **Baseline characteristics of children who underwent DBPCFC, n =191**

**Characteristic**	
Age, *mean (range) in yrs*	7.8 (3.4-18.6)
Male sex, *n (%)*	132 (70)
Peanut sIgE, *median (IQR) in kU/L*	2.60 (0.60-18.80)
Previous reaction to peanut, *n (%)*	
No ingestion / no reaction	96 (50)
Non severe	63 (33)
Severe	32 (17)
DBPCFC outcome, n (%)*	
Negative	103 (54)
Grade 1	2 (1)
Grade 2	51 (27)
Grade 3	15 (8)
Grade 4	20 (11)

*According to the Sampson classification of anaphylaxis [6].

Results of inter- and intra-observer analysis are shown in Table 3. The inter-observer agreement on overall food challenge outcome was moderate with κ = 0.59-0.65. Analysis of agreement in challenges with symptoms (n = 111) showed only fair agreement between observers, κ = 0.31-0.46. To assess intra-observer variability 48 (25%) randomly selected duplicated score sheets including 27 (14%) score sheets with reported symptoms could be used. The intra-observer agreement on overall challenge outcomes in duplicated challenges was, based on the kappa value, relatively good (κ = 0.63-0.77). The agreement within observers in challenges with symptoms (n = 27) was however moderate, κ = 0.37-0.60.

**Table 3:** **Agreement and variability in classification of DBPCFC outcome**

	**All DBPCFC (n =191)**			**DBPCFC with symptoms (n =111)**		
	**1**	**2**	**3**	**1**	**2**	**3**
**Agreement with initial DBPCFC outcome**	79%	82%	87%	65%	69%	78%
	**1:2**	**1:3**	**2:3**	**1:2**	**1:3**	**2:3**
**Inter-observer agreement**	78%	76%	76%	76%	61%	60%
**κ** *(95% CI)*	0.65 (0.56-0.74)	0.59 (0.50-0.68)	0.59 (0.50-0.68)	0.46 (0.39-0.53)	0.35 (0.22-0.48)	0.31 (0.25-0.38)
Overall agreement	69%			50%		
	**1**	**2**	**3**	**1**	**2**	**3**
Intra-observer agreement*	77%	83%	88%	67%	70%	81%
**κ** *(95% CI)*	0.63 (0.45-0.82)	0.71 (0.54-0.89)	0.77 (0.62-0.92)	0.50 (0.37-0.63)	0.52 (0.39-0.65)	0.60 (0.45-0.60)

DBPCFC, Double Blind Placebo Controlled Food Challenge.

κ, Kappa.

*n =48 (All DBPCFC) and n =27 (DBPCFC with symptoms).

**Bold** numbers express different observers. For example; **1** = observer 1 and **1:2** = observer 1 versus observer 2.

Clear objective symptoms (nasal and severe respiratory symptoms and urticaria) were associated with agreement whereas mild objective symptoms (mild respiratory symptoms, eye symptoms, sneezing and skin symptoms other than urticaria) and subjective signs and symptoms (OAS, abdominal complaints and food aversion) were associated with disagreement between observers (Table 4). The more different objective symptoms were present the more agreement between observers was observed (Table 4). The occurrence of subjective symptoms (e.g. abdominal complaints and OAS) was associated with disagreement within observers whereas disagreement was never present when respiratory symptoms occurred (data not shown). Four children (2%) experienced symptoms on a placebo portion during challenge, observers disagreed on challenge outcome in two of these children. Exclusion of children with placebo reactions did however not change the results of our study (data not shown).

**Table 4:** **Univariate association of symptoms during challenge with observer agreement, n =191**

**Tract**	**Symptoms**	**Disagree (n =59)**	**Agree (n =132)**	***p***
Upper airways	Red/itchy eyes	10 (17)	13 (10)	*0.168*
	Sneezing	10 (17)	10 (8)	*0.056*
	Nasal congestion/rhinorrhoea	-	10 (8)	0.043*
Lower airways	Cough	5 (9)	6 (5)	*0.282*
	Hoarseness/difficulty swallowing	-	3 (2)	*0.243*
	In- and/or expiratory stridor	-	5 (4)	***0.130***
	Wheezing	-	4 (3)	***0.177***
	Dyspnoea	3 (5)	2 (2)	*0.153*
Gastro-intestinal	OAS^	28 (48)	12 (9)	0.000**
	Abdominal complaints^	21 (36)	6 (5)	0.000**
	Vomiting	4 (7)	5 (4)	*0.374*
	Diarrhoea	-	-	-
Skin	Contact urticaria^#^	9 (15)	13 (9)	*0.283*
	Redness	12 (20)	9 (7)	0.008**
	Pruritis	4 (7)	6 (5)	*0.525*
	Urticaria	-	10 (8)	0.043*
	Angioedema	-	2 (2)	*0.342*
Neurological	Change in activity level/loss of consciousness	-	-	*-*
Other subjective signs	Discomfort^	2 (3)	10 (8)	*0.283*
	Food aversion^	14 (24)	10 (8)	0.003**
Number of different objective symptoms	No objective symptoms	18 (32)	2 (4)	*Ref*
	1 symptom	22 (39)	23 (40)	0.005**
	2 symptoms	13 (23)	22 (40)	0.001**
	3 symptoms	3 (5)	8 (15)	0.002**

OAS, Oral Allergy Symptoms; Ref, Reference category.

*^*Symptoms referred to as subjective symptoms.

^#^Local urticaria after direct contact between the challenge material and skin.

*Statistical significant association with agreement.

**Statistical significant association with disagreement.

**Bold** numbers are statistically significant (p <0.05).

## Discussion

Our results indicate that when presented with the same clinical information about symptoms during food challenges, clinical experts often (in more than 30%) disagree on food challenge outcome. While this fair amount of disagreement could be seen as disappointing, results could have been expected. It is known from previous studies in other disciplines that variability in interpretation of clinical symptoms is often present, despite the use of guidelines or scoring systems. Investigators of the Paediatric Rome II criteria for diagnosing functional gastrointestinal disorders in children showed low inter observer agreement among gastroenterologists (45% agreement, κ = 0.4), even when using a standardized symptom scoring system [7]. A study on the agreement between nurses who triaged patients presenting in the emergency room revealed only 52% agreement (κ = 0.3) [8]. Moreover a low level of agreement (κ = 0.3) among pediatric asthma specialists in classifying asthma serverity according to the NIH guidelines was found previously [9].

The origin of disagreement between and within observers observed in this study can be explained in several ways. Our results indicate that not the number but the origin and severity of symptoms is related to the amount of disagreement between observers. This is in contrast to previous suggestions that there is less room for doubt about challenge outcome when two or more organ systems are involved or when symptoms are reproducible or persisting [10]. Due to the amount of variability in course of symptoms during challenge between patients, we were unfortunately not able to demonstrate whether the timing of symptoms was related to the level of agreement between observers.

Subjective symptoms or mild objective symptoms (one episode of vomiting or a transient rash) frequently occur in children, usually as the first sign of an allergic reaction during food challenges. However these symptoms can also indicate fear associated with the clinical setting of the challenge or intolerance for the amount of food or the matrix chosen. As mainly subjective symptoms were present in cases on which observers disagreed one could argue that observers have difficulties in the interpretation of food challenge outcome when clear objective symptoms are absent. Moreover, guidelines only provide information on symptoms likely to be associated with positive challenge outcome and can therefore be interpreted and implemented by each observer differently. Reliability of the assessment of food challenges outcome also depends on the information provided. In our study lack of knowledge of the guidelines is unlikely to influence the results as all observers were clinical experts in the field of food allergy and used to perform and interpret food challenges. The same clinical information was administered to all observers excluding the possibility of sampling error. Assessment of challenge outcome was based on paper score sheets eliminating the possibility that the interpretation of observers and results of this study were influenced by other (patient related) factors as level of sensitization, age or previous challenge results.

To our knowledge this is the first study exploring agreement between clinical experts in assessing food challenge outcome. Observers reassessed a large number of challenges in a blinded, standardized and accurate way. Due to the retrospective nature there are some limitations that should be considered when interpreting the results of this study. Placebo reactions can influence challenge outcome in young children [11]. DBPCFCs were performed with randomly interspersed placebo’s, but observers had only access to blinded score sheets. Unfortunately we were therefore not able to analyse differences between placebo or verum challenges. Challenges were reassessed after two years, based on recorded symptoms during challenge, no additional (photographic or real life) patient information was available. The food challenges score sheet was not validated and lack of information could have caused differences between observers. Based on our results we can therefore not conclude that observers would classify challenge outcome of actual patients in the same manner as they did based on paper score sheets. However it is possible that the lack of agreement we found is even an underestimation of variability in assessment of ‘real life’ challenges since conditions in this study were standardized in contrast to real life reactions where observers are influenced by many other (patient related) factors.

## Conclusion

Although our study using symptom score sheets might not fully reflect procedures in a real life setting, our observations indicate that different observers may have different opinions about symptoms during food challenge tests. To further investigate whether this variability is also present during real life challenges future prospective studies using an expert panel or for example a scoring system with weightage points for each (type of) symptom to assess food challenge outcomes are needed. To improve standardization of food challenges and diminish variability in interpretation new preferably objective parameters might also be helpful in the future [12-16]. Until now, clinicians should be aware that although experienced and familiar in working according to international guidelines variability in interpretation of food challenge outcome is present when reassessing score sheets of challenges, especially when objective symptoms are absent.

## Abbreviations

DBPCFC: Double blind placebo controlled food challenge
OAS: Oral allergy symptoms
